# Environmental Adaptation Differences Are Key Factors Determining the Speciation and Future Adaptability of the Five Closely Related Species of the Genus *Ophioglossum*

**DOI:** 10.1002/ece3.73243

**Published:** 2026-03-12

**Authors:** Zhen‐Yan Pan, Jing‐Zhong Chen, Fu‐Li Gong, Shun‐Chao Jia, Qing‐Wen Sun

**Affiliations:** ^1^ College of Pharmacy Guizhou University of Traditional Chinese Medicine Guiyang P.R. China; ^2^ Guizhou Traditional Chinese Medicine National Medicinal Materials Germplasm Resources Preservation and Evaluation Engineering Research Center Guiyang P.R. China; ^3^ Guizhou Key Laboratory for Raw Material of Traditional Chinese Medicine Guiyang P.R. China

**Keywords:** environmental factors, GARP model, MaxEnt model, *Ophioglossum*, potential geographical distribution

## Abstract

*Ophioglossum* is an herbaceous genus of the Ophioglossaceae family with notable medicinal value and ecological importance in forest ecosystems. However, the impact of rising global temperatures on its distribution and the habitat ranges of its species in China remains unclear. This study assessed the potential distributions of five closely related *Ophioglossum* species under current and future climates, identified key environmental drivers, and compared the predictive performance of MaxEnt and GARP models to support conservation planning. Distribution models were built using occurrence records and environmental variables, with key factors identified through jackknife tests and response curves; performance was evaluated using AUC. Future projections were based on CMIP6 scenarios (SSP126, SSP245, SSP370, SSP585). Both models showed good predictive ability (AUC > 0.84), with MaxEnt demonstrating higher efficiency and stability. Species exhibited distinct environmental preferences: *O. petiolatum* responded mainly to temperature range and vapor pressure; *O. pedunculosum* to solar radiation and wind; *O. vulgatum* to vapor pressure and seasonal precipitation; *O. reticulatum* to solar radiation and diurnal temperature variation; and *O. thermale* to precipitation seasonality and soil properties. High‐suitability areas are currently concentrated in Yunnan, Sichuan, Guizhou, and Hunan provinces. Future changes in suitable habitat were limited, with no significant differences between the 2050s and 2070s. Climate change is not the primary near‐term driver of distribution shifts for these species; greater threats arise from habitat fragmentation, human disturbance, and reproductive constraints. Conservation efforts should prioritize enhancing habitat connectivity and protecting microhabitats in existing high‐suitability regions. This study provides a scientific basis for *Ophioglossum* conservation, and future work should incorporate population dynamics and dispersal mechanisms to improve predictive realism.

## Graphical abstract

This study employs MaxEnt and GARP models to assess the impact of climate change on five closely related *Ophioglossum* species in China, which are primarily distributed southwest of the Qinling Mountains. Key climatic drivers, particularly precipitation and water vapor pressure, significantly influence their potential ranges, with most species' suitable habitats projected to expand under future scenarios while one is expected to contract. The findings provide a critical foundation for the conservation and sustainable management of this medicinally and ecologically important genus.

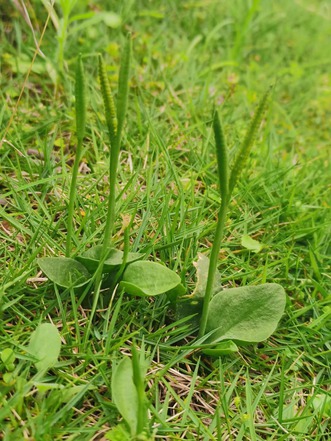

## 1 Introduction

The composition of plant species is the fundamental determinant of community structure and biodiversity conservation (Cubino et al. 2022; Thomas et al. 2023), though the influence of specific functional groups varies considerably (Wang et al. 2022). Among these, medicinal plants—defined by their richness in bioactive primary and secondary metabolites—constitute a functionally significant component of forest ecosystems (Mykhailenko et al. 2025). Beyond their pharmacological value, they maintain ecological balance, support environmental integrity (Li et al. 2025; Mbelebele et al. 2024), and critically shape ecosystem structure to sustain diversity and stability (Campos and Albuquerque 2020). These plants deliver essential biodiversity‐based ecosystem services, particularly health benefits, making their conservation imperative for sustainability (Nankaya et al. 2021). In view of their significance, researchers have repeatedly concentrated on characterizing the regional distribution of these species (Grimmett et al. 2021; Harisena et al. 2021).

While microclimatic niche partitioning is known to drive fern diversification (Nitta et al. 2020), empirical tests in closely related species are rare. To address this, we examined five *Ophioglossum* species that co‐occur across elevation gradients but differ in leaf traits and desiccation tolerance. Species of the medicinal fern genus *Ophioglossum* have long been utilized in traditional medicine for their anti‐inflammatory and antimicrobial properties (Yousaf et al. 2024). However, sustainable utilization and conservation of these resources are hindered by limited knowledge of their distribution dynamics and genetic diversity. Here, we selected five taxonomically problematic species ( *O. petiolatum* , *O. pedunculosum*, *O. vulgatum* , *O. reticulatum* , and *O. thermale*) that are increasingly threatened by habitat fragmentation. By integrating field surveys and molecular data, this study aims to delineate their biogeographic patterns and identify priority areas for conservation, thereby bridging the gap between ethnobotanical value and evidence‐based protection strategies.

At macroscales, climate dominates species distribution patterns (O'Connor et al. 2019; Woodward and Lomas 2004). Climate change, interacting with regional variability, reshapes ecosystems and species ranges (Shiqi et al. 2025; He and Silliman 2019). Combined with anthropogenic pressures, it drives habitat fragmentation, biodiversity loss (Schmeller and Bridgewater 2016), range contractions, and heightened extinction risks (Urban 2024; Urban et al. 2024; Pecl et al. 2017). Although localized disturbances may transiently increase richness during climatic transitions (Suggitt et al. 2019), rising temperatures elevate extreme weather and wildfire frequency. Moisture‐stressed forests face increased flammability, challenging plant establishment and distribution (Cui et al. 2020; Zhang, Yang, et al. 2023; Zhang, Zhao, et al. 2023; Mansoor et al. 2021). Projections indicate significant range contractions for many species, with documented shifts toward higher elevations and latitudes (Hamann and Wang 2006; Wang et al. 2019; Prasad et al. 2020; Yuan et al. 2022; Parmesan and Yohe 2003), though counterexamples exist (Su, Bista, and Li 2021; Su, Huang, et al. 2021; Tagliari et al. 2021; Zorio et al. 2016). Beyond climate, edaphic factors, topography, and human activities critically regulate distributions (Liu et al. 2020; Tian et al. 2019). Elevational gradients modulate thermal regimes, moisture, and soil nutrients, acting as primary distribution filters (Oke and Thompson 2015; Thakur and Chawla 2019). Human activities exert complex, often opposing effects on species ranges (Prugh 2023; Bell et al. 2022), with climate‐anthropogenic synergies impairing plant growth, reproduction, and medicinal resource quality (Mykhailenko et al. 2025).

Ecological Niche Models (ENMs), also known as Species Distribution Models (SDMs), is pivotal tools for projecting climate‐driven range shifts (Wittmann et al. 2016; Isaac et al. 2019). These algorithms correlate species occurrences with environmental variables to predict potential distributions (Laxton et al. 2022; Lomolino 2023). ENMs identify habitats (Chakraborty et al. 2016; Dallas and Hastings 2018), assess range dynamics and extinction risks (Mariángeles et al. 2025; Ahmadi et al. 2019), and evaluate biodiversity under future scenarios (Poggiato et al. 2021; Quiroga and Souto 2022). Common approaches include climate‐envelope methods (Bioclim) (Booth et al. 2013), similarity‐based frameworks (Domain) (Duan et al. 2014), experimental simulators (Climex) (Souza et al. 2023), and ensemble platforms (Biomod2) integrating machine learning (e.g., RF, GLM, GAM) (Yang et al. 2025; Yisen et al. 2017; Zhang, Chen, et al. 2016; Zhang, Yu, et al. 2016; Barna et al. 2023). The robust Maximum Entropy (MaxEnt) (Warren and Seifert 2011) and Genetic Algorithm for Rule‐set Prediction (GARP) (Stockwell 1999) models excel with presence‐only data, especially for data‐limited or range‐restricted species (Byeon et al. 2018; Merow et al. 2013). Both demonstrate superior predictive accuracy (Su, Bista, and Li 2021; Su, Huang, et al. 2021) and are widely applied in biogeography (Gomes et al. 2018; Morera‐Pujol et al. 2022) and invasion biology (Wang et al. 2010). Ensemble approaches integrating multiple SDMs reduce uncertainty and enhance reliability (Araújo and New 2006; Srivastava et al. 2018).

Given the unclear habitat trajectories of Chinese medicinal plants under climate change, and based on the known ecological preferences of *Ophioglossum* species and observed responses of plants to warming, we hypothesized that (1) the distributions of these five closely related species are driven by distinct climatic factors; (2) future climate change will shift their suitable habitats toward higher elevations and latitudes; and (3) interspecific differences in environmental adaptability will lead to divergent distributional responses among species. To test these hypotheses, we modeled the potential distributions of five *Ophioglossum* species using MaxEnt and GARP under current and future climates. Our objectives were to (1) predict the potential geographical distribution of five *Ophioglossum* species in current and future climates using MaxEnt and GARP models; (2) identify the key environmental driving factors affecting the distribution of each species; (3) compare the change trend of suitable areas under different climate scenarios; and (4) explore the adaptive differences between species and their response mechanisms to climate change. Results will inform conservation strategies essential for maintaining ecosystem stability.

## 2 Materials and Methods

### 2.1 Species Occurrence Point Data

**FIGURE 1:**
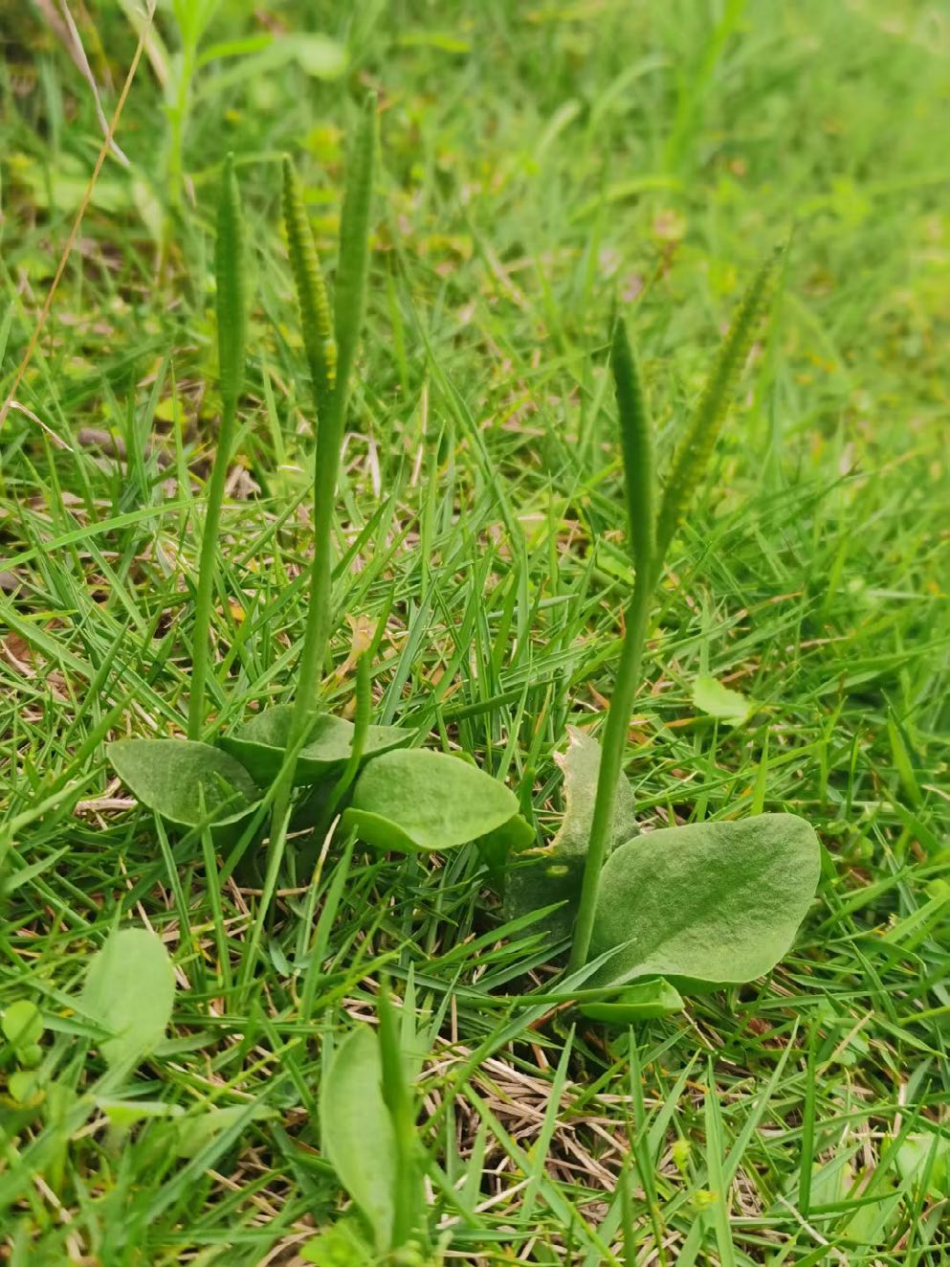
Morphological characteristics of representative *Ophioglossum* plants ( *O. vulgatum* ).

Occurrence data for the five *Ophioglossum* species were compiled from the Global Biodiversity Information Facility (GBIF, https://www.gbif.org/zh), Chinese Virtual Herbarium (CVH, https://www.cvh.ac.cn), and Plant Photo Bank of China (PPBC, http://ppbc.iplant.cn), supplemented by field surveys. Initial records totaled 40 ( *O. petiolatum* ), 53 (*O. pedunculosum*), 261 ( *O. vulgatum* ), 107 ( *O. reticulatum* ), and 50 (*O. thermale*). The morphological characteristic of the representative species of this genus, *O. vulgatum* , is shown in Figure 1. The images were taken by Professor Qing‐Wen Sun, and their use in this study has been authorized by him in strict compliance with relevant copyright regulations. For occurrences lacking coordinates but with precise locality descriptions (township‐level or finer), we georeferenced points using the Coordinate Picking System (https://jingweidu.bmcx.com) and compiled data in CSV format. Specimens with vague locality data were excluded. To minimize spatial autocorrelation, we retained only one occurrence per 10 × 10 km grid cell in ArcGIS (Wu et al. 2024). Following removal of erroneous coordinates, low‐precision records, and spatial duplicates, we obtained 37 ( *O. petiolatum* ), 43 (*O. pedunculosum*), 212 ( *O. vulgatum* ), 92 ( *O. reticulatum* ), and 43 (*O. thermale*) validated occurrences (Table S1). Chinese distribution maps were generated using these coordinates in ArcGIS 10.8 (Figures S1 and S2).

### 2.2 Environmental Factor Data

Three environmental variable categories were selected: climatic, edaphic, and topographic factors (Table S2). Climatic variables (2.5 arc‐minute resolution) were sourced from WorldClim2.1 (https://worldclim.org/), including baseline (1970–2000), mid‐century (2041–2060), and late‐century (2061–2080) periods. For future climate data, we employed the BCC‐CSM2‐MR model under the Shared Socioeconomic Pathways (SSPs) scenarios released by the Intergovernmental Panel on Climate Change (IPCC) in its Sixth Assessment Report (AR6), “Climate Change 2021: The Physical Science Basis”, which is based on the Sixth Phase of the Coupled Model Intercomparison Project (CMIP6). The simulated precipitation and temperature patterns of this model have been shown to align well with observed conditions in China (Li et al. 2023). BCC‐CSM2‐MR's enhanced atmospheric resolution and topographic representation improve simulations of extreme temperatures, precipitation patterns, and local climate dynamics (Wu et al. 2019). Four scenarios were evaluated: SSP126 (low radiative forcing), SSP245 (intermediate), SSP370 (medium‐high), and SSP585 (high forcing), representing increasing greenhouse gas concentrations.

Edaphic data (1 km resolution) came from the China Soil Properties Dataset for Land Surface Modeling v2 (CSDLv2; National Tibetan Plateau Data Center: https://data.tpdc.ac.cn). Topographic variables (1 km resolution) were derived from the China Digital Elevation Model (DEM: https://data.tpdc.ac.cn). Twenty‐seven soil and three topographic predictors were selected based on the ecological requirements of *Ophioglossum* species and factor relevance. Additionally, environmental factor data for China were extracted using Geographic Information System software (ArcGIS). All environmental data layers were standardized to the WGS_1984 coordinate system and resampled to a uniform resolution of 2.5 arc‐minute.

The vector map of China was obtained from the public‐service map (Approval No. GS (2020) 4619) provided by the National Geomatics Center of China.

### 2.3 Setting of MaxEnt and GARP Models

We used MaxEnt 3.4.4 to model suitable habitats for the five species of *Ophioglossum*. Occurrence records and environmental variables were imported into the model, with 25% of points reserved for testing, a background sample size of 10,000, and 10 bootstrap replicates; other parameters were left at default settings (Mohadeseh et al. 2022; Wu et al. 2024). The choice of using 25% for testing and 75% for training was further validated through spatial cross‐validation. The results are presented in Table S3. Model tuning was performed in R 4.1.3 using the ENMeval package. For the 5 species linear characterization of the FC parameters: linear (L), quadratic (Q), hinge (H), product (P) and threshold (T), and we tested six feature‐class (FC) combinations (L, LQ, H, LQH, LQHP, LQHPT) across regularization multipliers (RM) ranging from 0.5 to 4 in steps of 0.5. The model with the lowest corrected Akaike information criterion (AICc) was selected as optimal (Phillips et al. 2017). The final selected parameters are summarized in Table S4. Within MaxEnt, we enabled “Create response curves” and “Do jackknife to measure variable importance” to assess the influence of individual environmental variables on predicted habitat suitability.

Desktop GARP v1.1.6 was implemented with 70% of occurrences for training and 30% for validation. Model parameters included 100 iterations, a 1000‐iteration maximum, and a 0.01 convergence limit (Anderson et al. 2003). Environmentally screened variables from MaxEnt were input to GARP. The algorithm integrates four rule types: atomic, range, negated range, and logistic regression. Rule combinations (*n* = 15) underwent 100 iterations each (1500 total). Optimal rules were selected by minimizing the average of extrinsic omission error [Omission(ext)] and intrinsic omission error [Omission(int)] (Table S5). Subsequent jackknife testing omitted individual variables across 100 runs per exclusion. Variables whose omission reduced prediction error beyond the 95% confidence interval of baseline error were deemed non‐significant and removed (Wang and Wang 2006).

### 2.4 Filters of Environmental Factors

Three environmental variable categories were analyzed: climatic, edaphic, and topographic factors. To prevent overfitting and enhance model accuracy, we implemented a multi‐step screening process. Firstly, initial contribution screening: All 134 environmental variables and the five species of *Ophioglossum* occurrence data were processed in MaxEnt. Variables with zero contribution were iteratively removed until all retained predictors exhibited non‐zero contribution. Secondly, correlation analysis: Remaining variables were extracted for occurrence locations using ArcGIS. Pairwise Pearson correlation coefficients were calculated in SPSS26. When |*r*| ≥ 0.8, the variable with lower MaxEnt contribution was excluded (Wang et al. 2024). The final environmental variables for the five species are listed in the table below (Table S6). Finally, final modeling: The screened variable set was used in 10 replicated MaxEnt runs. Habitat suitability was assessed using mean output values. After successful simulation, we used climatic factors related to the prediction period when predicting the suitable habitat distribution of species under different climate scenarios in the future, in which soil and topographic factors remained unchanged (Guo et al. 2018).

The environmental variables used in GARP were the final subset selected after preliminary screening and collinearity testing with MaxEnt, ensuring a consistent basis for comparison between the two models.

### 2.5 Model Evaluation

The AUC value is a widely recognized metric for assessing model performance (Neftalí et al. 2021). The ROC metric is determined by computing the area under the curve (AUC). AUC refers to the area under the ROC (receiver operating characteristic) curve, which is usually utilized for testing the accuracy of a model, and it is not affected by the proportion of subjects in the analyzed sample (Parodi et al. 2022). AUC was frequently used in the evaluation of the performance of a variety of models for the Species Distribution Model (SDM) and is not impacted by the threshold setting. In this research, the magnitude of AUC values was utilized to assess the predictive effectiveness of respective models. Larger AUC values indicate greater correlation between the modeled geographic distribution of target species and environmental elements, indicating that the predictive performance of this model is better (Ma et al. 2021). For the model, the prediction accuracy was categorized into five levels: fail (0.5–0.6), poor (0.6–0.7), fair (0.7–0.8), good (0.8–0.9), and excellent (0.9–1) (Zhao et al. 2024). The accuracy is high, and the potential distribution state of the reaction species can be more accurately reflected.

### 2.6 Suitability Classification

Habitat suitability outputs from MaxEnt and GARP were imported into ArcGIS 10.8 in ASCII format, georeferenced to the GCS_WGS_1984 coordinate system, and visualized as potential distribution maps. To convert continuous suitability probabilities (P, ranging from 0 to 1) into binary suitable/unsuitable areas, we applied the Maximum Test Sensitivity Plus Specificity (MTSS) threshold—a widely accepted and ecologically interpretable approach in species distribution modeling. For clearer ecological interpretation, suitability was further classified into four levels: unsuitable (*P* ≤ MTSS), low suitability (MTSS < *p* ≤ 0.4), medium suitability (0.4 < *p* ≤ 0.6), and high suitability (*p* > 0.6) (Mahatara et al. 2021; Aidoo et al. 2022).

## 3 Results

### 3.1 Model Accuracy Test

In this study, the AUC was selected as the basis for the assessment of model accuracy. We respectively obtained AUC values of 0.914, 0.881, 0.884, 0.906, and 0.872 for the MaxEnt model of the five closely related species of *Ophioglossum* (Figure S3). The AUC values of the GARP model for 10 replicates were 0.861, 0.966, 0.884, 0.941, and 0.846, indicating that the performance of the distribution model was better than that of the random model, and the stability between each repetition was good. The model had good and excellent performance in predicting the suitable area of *Ophioglossum*.

### 3.2 Influence of Major Environmental Factors

**FIGURE 2:**
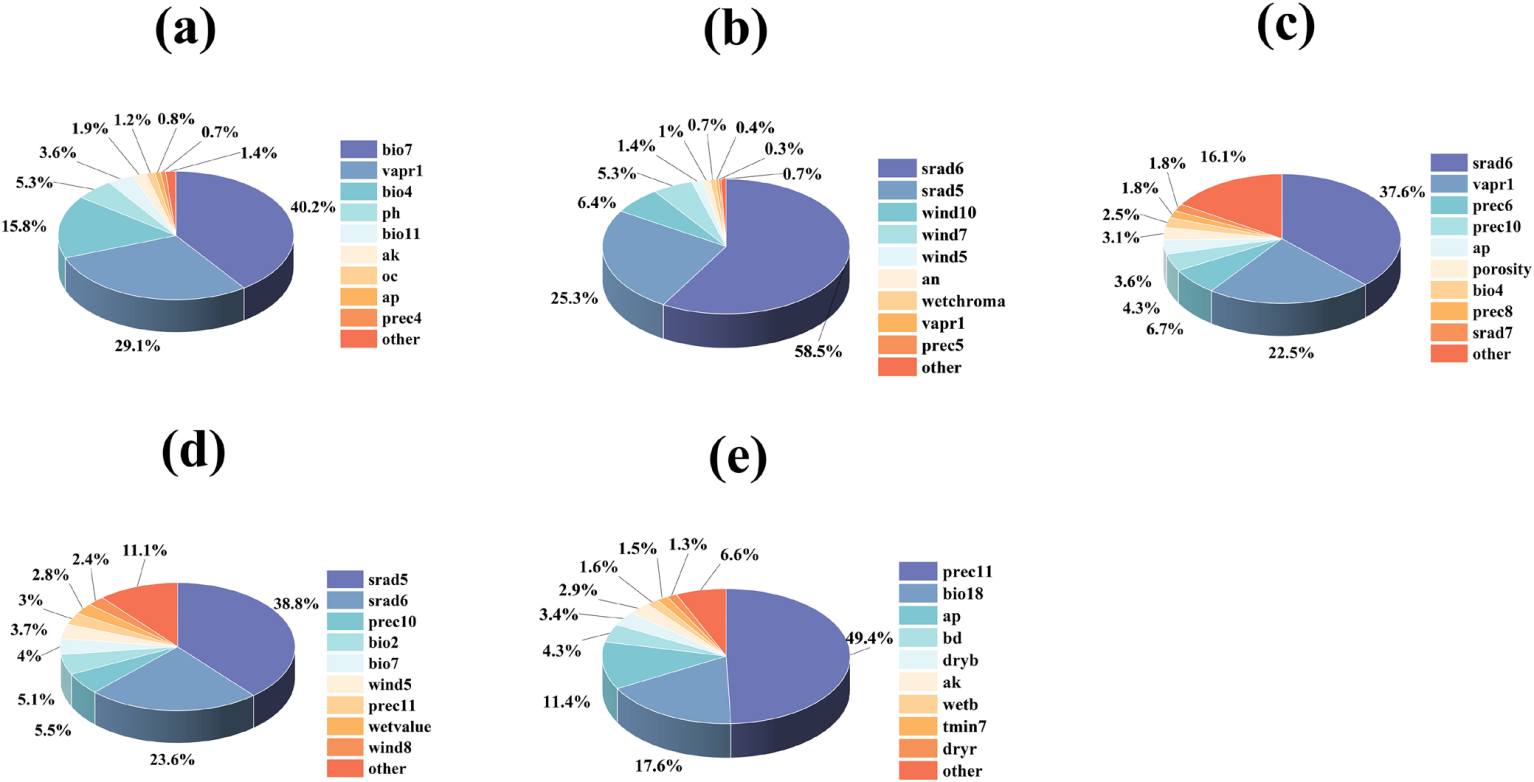
Importance ranking of environmental variables used for (a) *O. petiolatum* , (b) *O*. *pedunculosum*, (c) *O. vulgatum* , (d) *O. reticulatum* , and (e) *O*. *thermale* tested by the pie chart method.

Variable importance for the five *Ophioglossum* species was evaluated using jackknife tests (Figure S4) and summarized in Figure 2. *O. petiolatum* was primarily influenced by the annual temperature range (bio7, 40.2%), followed by January water vapor pressure (vapr1, 25.1%), temperature seasonality (bio4, 15.8%) and soil pH (ph, 5.3%). *O. pedunculosum* showed strong dependence on solar radiation in June (srad6, 58.5%) and May (srad5, 25.3%), with minor contributions from wind speed in October (wind10, 6.4%) and July (wind7, 5.3%). For *O. vulgatum* , June solar radiation (srad6, 23.2%) and January vapor pressure (vapr1, 19.3%) were the most important predictors, accompanied by precipitation in the wettest month (prec6, 6.7%) and driest month (prec10, 4.3%). *O. reticulatum* was most sensitive to May solar radiation (srad5, 38.8%) and June solar radiation (srad6, 23.6%), followed by October precipitation (prec10, 5.5%) and diurnal temperature range (bio2, 5.1%). *O. thermale*'s suitability was largely explained by November precipitation (prec11, 30.4%), precipitation seasonality (bio18, 17.6%), available phosphorus (ap, 11.4%), and bulk density (bd, 4.3%).

**FIGURE 3:**
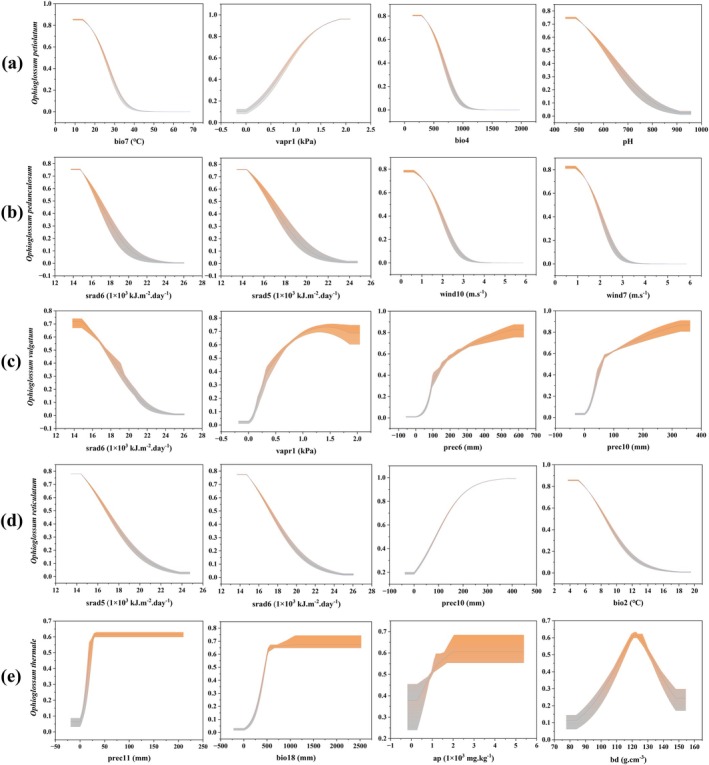
Response curves for important environmental predictors in the species distribution model of (a) *O. petiolatum* , (b) *O*. *pedunculosum*, (c) *O. vulgatum* , (d) *O. reticulatum* , and (e) *O*. *thermale*. The horizontal coordinate represents the magnitude of the environmental predictors, and the value of the vertical coordinate indicates the occurrence probability of the foundation species.

The response curves and environmental ranges revealed distinct niche preferences among the five *Ophioglossum* species (Figure 3, Table S7). *O. petiolatum* exhibited a pronounced unimodal response to bio7, with suitability peaking around 15°C–20°C, and a broad tolerance for vapr1. Its distribution was further associated with relatively high soil pH. In contrast, *O. pedunculosum* and *O. reticulatum* both showed strong positive responses to high srad5 and srad6, but *O. reticulatum* was restricted to sites with lower bio2 (bio2 < 9°C). *O. vulgatum* displayed a wider vapr1 niche (0.57–2.05 kPa) and a monotonic increase in suitability with prec6. *O. thermale* was uniquely associated with pronounced bio18 and specific soil conditions—its suitability rose sharply with ap up to more than 3000 mg·kg^−1^ and showed a narrow optimum for bd.

### 3.3 Distribution of Suitable Areas for the Five Closely Related Species of *Ophioglossum* Under Current Climate Conditions

**FIGURE 4:**
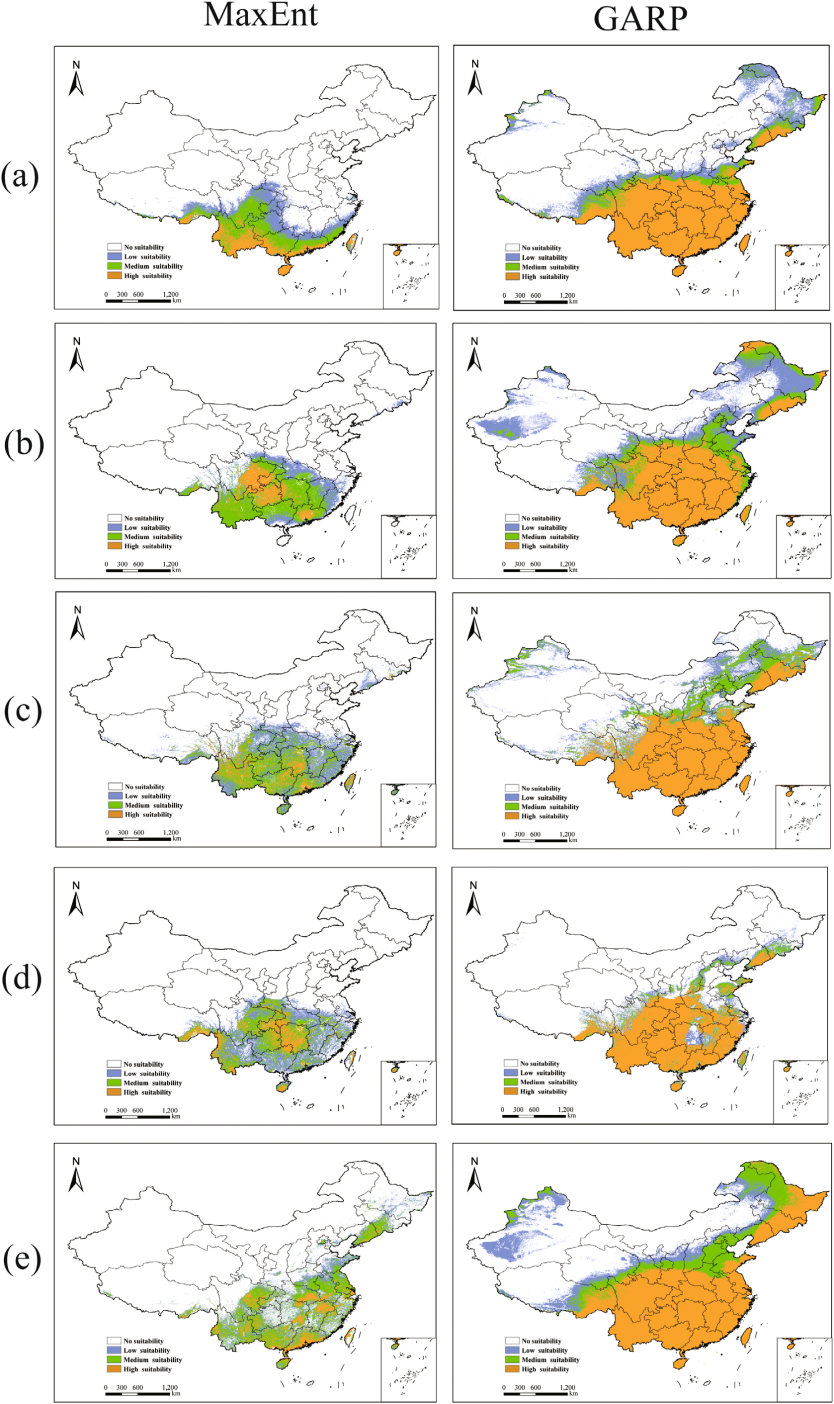
Under the MaxEnt model and GARP model, current habitat ranges of the (a) *O. petiolatum* , (b) *O*. *pedunculosum*, (c) *O. vulgatum* , (d) *O. reticulatum* , and (e) *O*. *thermale* in China. Legend colors indicate the occurrence probability, including four suitability levels: No suitability (white), low‐suitability zones (purple), moderate‐suitability zones (green), and high‐suitability zones (orange).

Under current climatic conditions, predictions of potential geographic distributions for the five *Ophioglossum* species from MaxEnt and GARP models were broadly consistent at a macro‐scale but showed clear differences in spatial extent and local boundaries (Figure 4 and Table 1). MaxEnt generally projected more concentrated suitable areas focused in central‐southern China, whereas GARP predicted broader expansion, particularly extending toward eastern and northeastern regions.

**TABLE 1:** Area proportion of potential suitable habitats for five *Ophioglossum* species predicted by MaxEnt and GARP models.

Suitability	*O. petiolatum*		*O. pedunculosum*		*O. vulgatum*		*O. reticulatum*		*O. thermale*	
	MaxEnt	GARP	MaxEnt	GARP	MaxEnt	GARP	MaxEnt	GARP	MaxEnt	GARP
No suitability (%)	82.42	53.84	80.15	44.06	77.13	50.09	78.58	67.90	76.68	39.15
Low suitability (%)	6.26	9.90	4.95	15.32	8.82	9.27	8.55	5.28	5.99	11.37
Medium suitability (%)	7.19	6.26	10.84	12.11	10.98	10.05	8.87	4.67	13.05	13.56
High suitability (%)	4.13	30.00	4.06	28.51	3.07	30.59	4.00	22.15	4.28	35.92
Potential suitability (%)	17.58	46.16	19.85	55.94	22.87	49.91	21.42	32.10	23.32	60.85

From the MaxEnt results, the core high‐suitability zones exhibited distinct regional patterns: *O. petiolatum* (17.58% of total national land area) was concentrated in Hainan, Taiwan, Yunnan, Guangxi, Guangdong, and neighboring provinces; *O. pedunculosum* (19.85%) was primarily distributed in Sichuan, Guizhou, Chongqing, and Guangdong; *O. vulgatum* (22.87%) was scattered across multiple southern provinces from Yunnan to Taiwan; *O. reticulatum* (21.42%) was mainly located from Yunnan and Guizhou to Hainan; and *O. thermale* (23.32%) was widespread in Guangdong, Hubei, Jiangxi, and adjacent areas.

The suitable ranges projected by GARP were markedly larger than those from MaxEnt, with highly similar distribution patterns across species covering most of central‐southern and northeastern China. Specifically, the total suitable areas for *O. petiolatum* , *O. pedunculosum*, *O. vulgatum* , *O. reticulatum* , and *O. thermale* accounted for 46.16%, 55.94%, 49.91%, 32.10%, and 60.85% of the national land area, respectively. The high‐suitability class generally exceeded 20%, reaching 35.92% in *O. thermale*, indicating that GARP tends to estimate broader ecological niche widths.

### 3.4 Distribution and Range Shifts in China for Five Species of *Ophioglossum* Under the Future Climate Change Scenarios

**FIGURE 5:**
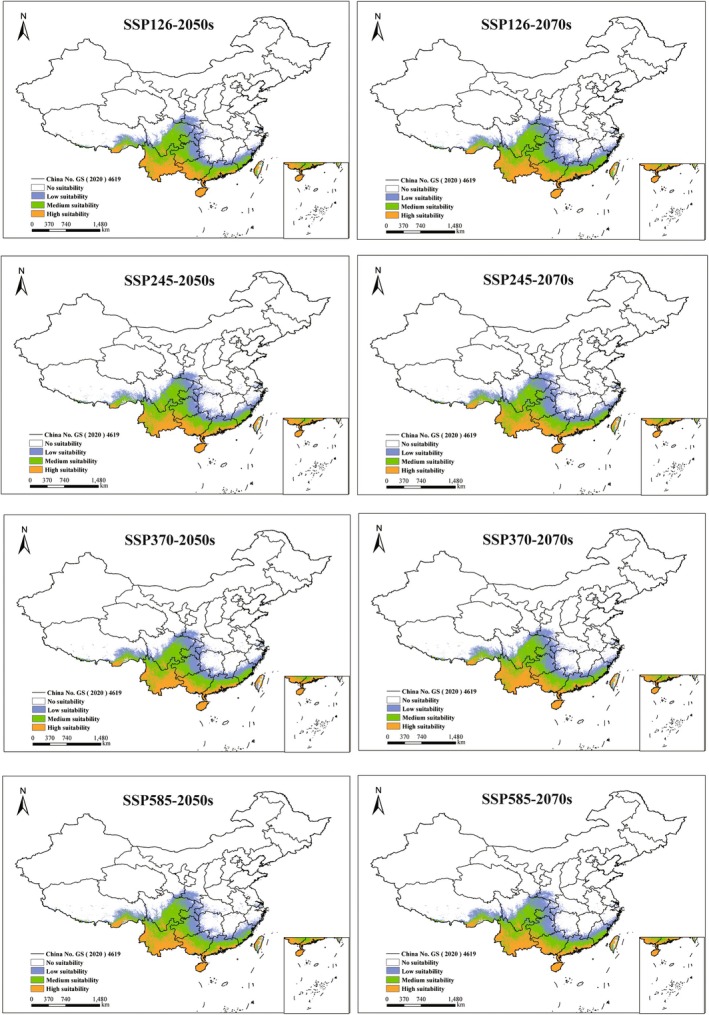
Changes in potentially suitable habitats for *O. petiolatum* projected by MaxEnt under four future climate scenarios (2050s and 2070s). (No suitability: white, low‐suitability zones: green, moderate‐suitability zones: purple, and high‐suitability zones: orange.)

**FIGURE 6:**
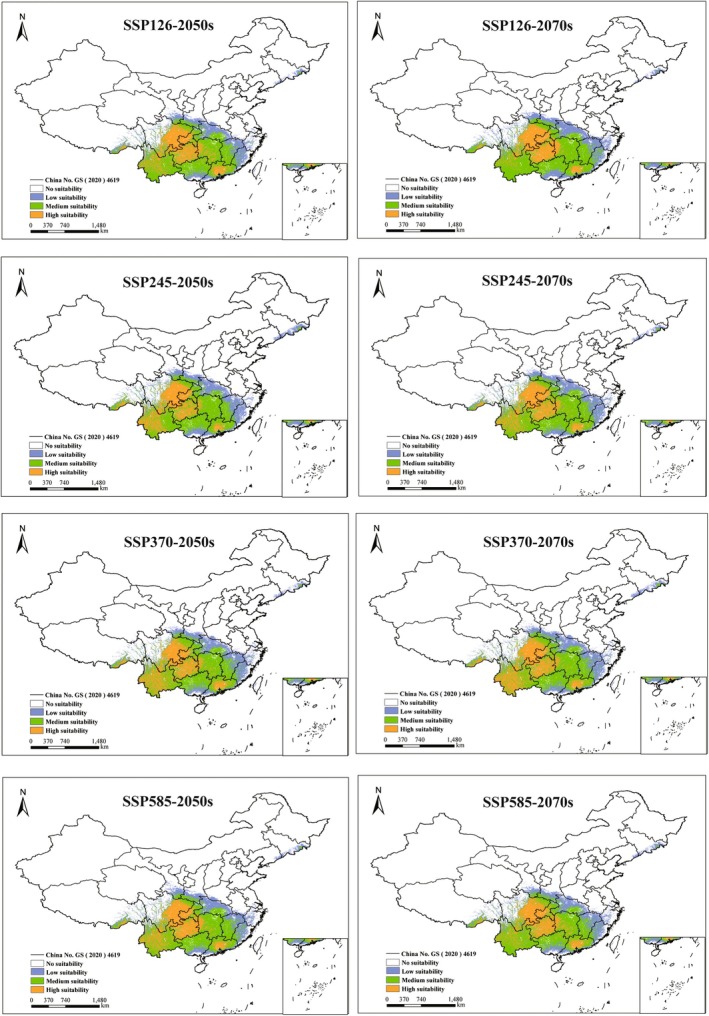
Changes in potentially suitable habitats for *O*. *pedunculosum* projected by MaxEnt under four future climate scenarios (2050s and 2070s). (No suitability: white, low‐suitability zones: green, moderate‐suitability zones: purple, and high‐suitability zones: orange.)

**FIGURE 7:**
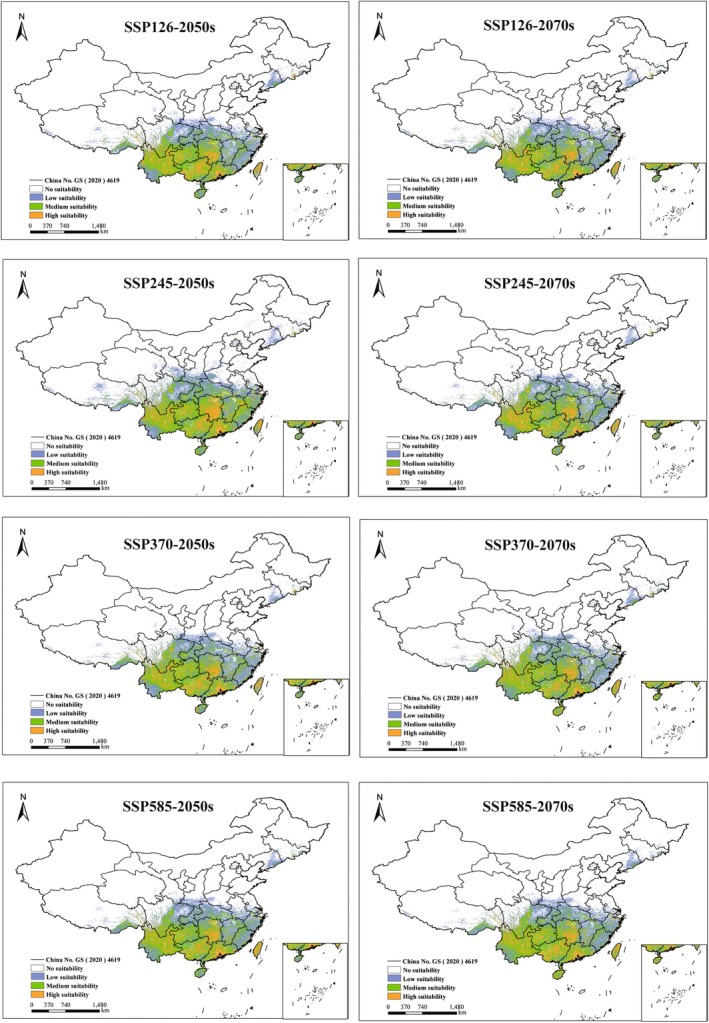
Changes in potentially suitable habitats for *O. vulgatum* projected by MaxEnt under four future climate scenarios (2050s and 2070s). (No suitability: white, low‐suitability zones: green, moderate‐suitability zones: purple, and high‐suitability zones: orange.)

**FIGURE 8:**
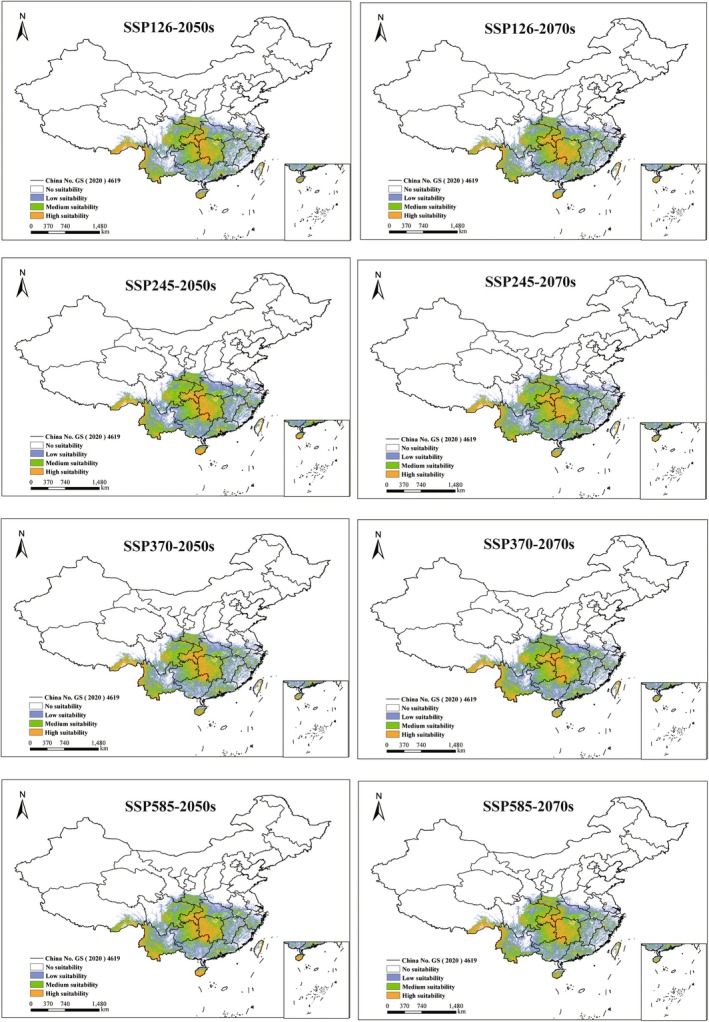
Changes in potentially suitable habitats for *O. reticulatum* projected by MaxEnt under four future climate scenarios (2050s and 2070s). (No suitability: white, low‐suitability zones: green, moderate‐suitability zones: purple, and high‐suitability zones: orange.)

**FIGURE 9:**
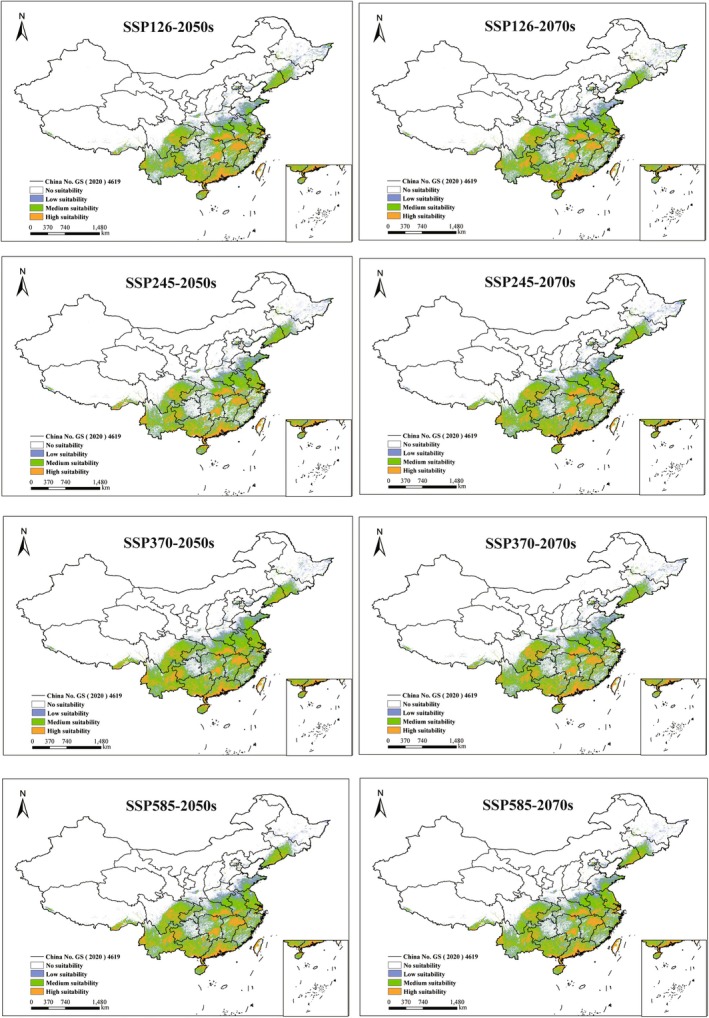
Changes in potentially suitable habitats for *O*. *thermale* projected by MaxEnt under four future climate scenarios (2050s and 2070s). (No suitability: white, low‐suitability zones: green, moderate‐suitability zones: purple, and high‐suitability zones: orange.)

Under identical climate scenarios, the proportional coverage of potentially suitable habitats for the five *Ophioglossum* species showed no significant differences between the 2050s and the 2070s (Figures 5, 6, 7, 8, 9). However, species‐specific and scenario‐dependent variations in area proportion were observed relative to current distributions (Table 2).

**TABLE 2:** Changes in proportional area of potentially suitable habitats for five *Ophioglossum* species under current and future climate scenarios projected by MaxEnt (“−” indicates decline relative to current extent).

Species	Suitability	Current	Future 2050s (2041–2060)				Future 2070s (2061–2080)			
			ssp126	ssp245	ssp370	ssp585	ssp126	ssp245	ssp370	ssp585
*O. petiolatum*	No suitability (%)	82.42	0.64	0.28	−0.03	−0.05	−0.58	0.13	0.18	0.32
	Low suitability (%)	6.26	−0.81	−0.29	−0.09	−0.30	−0.12	−0.16	−0.48	−0.17
	Medium suitability (%)	7.19	0.04	0.02	−0.09	0.00	0.25	−0.09	−0.14	−0.37
	High suitability (%)	4.13	0.13	−0.01	0.22	0.35	0.46	0.13	0.44	0.21
*O. pedunculosum*	No suitability (%)	80.15	−0.44	−0.34	−0.26	−0.81	−0.67	−0.07	−0.07	−0.44
	Low suitability (%)	4.95	0.00	0.23	0.09	0.23	0.23	0.02	0.02	0.13
	Medium suitability (%)	10.84	−0.43	−0.54	−0.89	−0.64	−0.03	−0.69	−0.69	−0.35
	High suitability (%)	4.06	0.87	0.64	1.07	1.23	0.47	0.74	0.74	0.66
*O. vulgatum*	No suitability (%)	77.13	0.21	−2.48	0.39	0.29	0.54	0.27	−0.02	0.06
	Low suitability (%)	8.82	0.16	0.23	−0.17	−0.19	0.31	0.25	0.02	−0.34
	Medium suitability (%)	10.98	−0.70	1.10	−0.55	−0.33	−1.04	−0.95	−0.22	−0.04
	High suitability (%)	3.07	0.33	1.14	0.33	0.23	0.20	0.43	0.22	0.32
*O. reticulatum*	No suitability (%)	78.58	1.23	0.36	0.81	0.52	0.77	0.33	0.84	0.79
	Low suitability (%)	8.55	−0.45	−0.31	−0.33	−0.20	−0.30	−0.20	−0.30	−0.45
	Medium suitability (%)	8.87	−0.68	0.07	−0.46	−0.25	−0.54	0.02	−0.46	−0.18
	High suitability (%)	4.00	−0.11	−0.12	−0.02	−0.07	0.07	−0.15	−0.08	−0.16
*O. thermale*	No suitability (%)	76.68	−0.35	0.18	−2.09	−1.43	−0.28	−1.90	−1.03	−1.17
	Low suitability (%)	5.99	0.03	−0.43	−0.92	−0.65	−0.15	−0.04	−0.19	−0.66
	Medium suitability (%)	13.05	0.31	0.17	2.24	1.43	0.61	1.63	0.76	1.38
	High suitability (%)	4.28	0.02	0.07	0.77	0.65	−0.18	0.31	0.46	0.45

*Ophioglossum petiolatum* exhibited both range contraction and expansion across future periods, with the most pronounced contraction (0.64%) occurring under SSP126 in the 2050s and the greatest expansion (0.58%) under the same scenario in the 2070s. *O. pedunculosum* consistently showed range expansion under all future scenarios, with increases ranging from 0.07% to 0.81%; the maximum increase occurred under SSP585 in the 2050s, while the minimum increases were observed under SSP245 and SSP370 in the 2070s.

*Ophioglossum vulgatum* displayed clear scenario‐dependent shifts, with the largest expansion (2.48%) under SSP245 in the 2050s and the most notable contraction (0.45%) under SSP126 in the 2070s. In contrast, *O. reticulatum* consistently underwent range contraction across all future scenarios, with losses ranging from 0.33% to 1.23%; the strongest contraction occurred under SSP126 in the 2050s, and the weakest under SSP245 in the 2070s.

*Ophioglossum thermale* expanded under nearly all scenarios except for a slight contraction (0.18%) under SSP245 in the 2050s. Its expansion peaked at 2.09% under SSP370 in the 2050s and reached a minimum of 0.28% under SSP126 in the 2070s.

## 4 Discussion

### 4.1 Model Performance Comparison: Divergent Outcomes Arising From Algorithmic Differences

This study employed the MaxEnt and GARP models to predict and compare the potential geographical distributions of five closely related species of the genus *Ophioglossum* (Moreno‐Amat et al. 2015). Model evaluation results indicated that the test AUC values for both models exceeded 0.84, demonstrating good predictive performance. However, their performance varied among species: the MaxEnt model showed better results for *O. pedunculosum* and *O. thermale*, whereas the GARP model achieved higher AUC values for *O. petiolatum* and *O. reticulatum* . These findings differ to some extent from previous studies that generally reported superior performance of MaxEnt over GARP (Fu et al. 2024; Padalia et al. 2014).

This discrepancy primarily stems from the different algorithmic principles and optimization objectives of the two models (Ray et al. 2017). The MaxEnt model, based on the maximum entropy principle, focuses on reducing the “commission error” and tends to produce more conservative distribution predictions (Phillips et al. 2006). Its results align more closely with the current distribution ranges documented in the *Flora of China* (Liu, Chen, et al. 2023; Liu, Zhao, et al. 2023; Zhang, Chen, et al. 2016; Zhang, Yu, et al. 2016). In contrast, the GARP model, which is based on a genetic algorithm rule set, emphasizes minimizing the “omission error” (Haase et al. 2021). Consequently, it often predicts broader potential geographical ranges but may be associated with a higher risk of false positives (Townsend Peterson et al. 2007; Zhu et al. 2017). After comprehensively considering the stability of the models' average AUC values and their computational efficiency (He et al. 2023; Zhang, Yang, et al. 2023; Zhang, Zhao, et al. 2023), the MaxEnt model was selected for future climate scenario projections in this study.

### 4.2 Ecological Niche Differentiation: Species‐Specific Responses to Environmental Gradients

Beyond the established dominant roles of temperature and precipitation (Dong et al. 2023), this study found that factors such as vapor pressure deficit, solar radiation, temperature seasonality, and soil properties further drive niche differentiation among the five *Ophioglossum* species. Each species exhibits a distinct environmental adaptation profile: *O. petiolatum* is mainly influenced by annual temperature range (bio7) and vapor pressure (vapr1); *O. pedunculosum* is sensitive to solar radiation (srad5, srad6) and wind speed (wind7, wind10), adapting to open, well‐ventilated, high‐light environments; *O. vulgatum* is closely associated with moisture conditions (vapr1, prec6, prec10); *O. reticulatum* relies on solar radiation and diurnal temperature variation (bio2); and *O. thermale* shows a significant dependence on precipitation seasonality (bio18) and soil physicochemical properties such as available phosphorus and bulk density.

These species‐specific environmental response patterns reveal that closely related taxa can evolve divergent adaptation strategies across multidimensional environmental gradients. Although the genus overall prefers shaded and moist habitats (Zhu et al. 2014), the prominent response of *O. thermale* to soil factors indicates that soil properties can further promote niche partitioning within the genus, which may be related to its spatial isolation and differential vulnerability. Such differentiation may be driven by evolutionary processes like allopatric speciation and local adaptation, where environmental heterogeneity shapes morphological and functional trait differences, thereby influencing species' geographical distributions and genetic structures (Antunes et al. 2018; Tarroso et al. 2014; Nicotra et al. 2016).

### 4.3 Conservation Implications Under Climate Change: Habitat Dynamics and Future Vulnerability

Predictions based on the MaxEnt model indicate that current high‐suitability areas for the five *Ophioglossum* species are primarily concentrated in Yunnan, Sichuan, Guizhou, and Hunan provinces. Among them, *O. thermale* has the widest potential geographical distribution, followed by *O. vulgatum* , whereas *O. petiolatum* has the smallest suitable area, accounting for only approximately 17.58% of China's land area. Its sporadic distribution in northeastern China aligns with relict and fragmented distribution characteristics (Yousaf et al. 2024; Zhang and Zheng 2000).

Under future climate scenarios, the impact of environmental changes on the suitable habitats of each species is generally limited, with no significant differences predicted between the 2050s and 2070s. This suggests that climate change may not be the primary direct driver of their near‐term distribution shifts. However, the projected stability could also be influenced by our use of a single climate model (BCC‐CSM2‐MR). Instead, habitat fragmentation, anthropogenic disturbances, and species‐specific limitations on reproduction and establishment (such as dependence on spore reproduction and requirements for specific shaded microhabitats and soil microbial symbionts) are likely more critical factors affecting their population persistence (Brock 2025; Dinh et al. 2023). Therefore, conservation efforts should focus on current high‐suitability areas by enhancing habitat connectivity and protecting key microhabitats to mitigate threats such as overharvesting, pests, and wildfires (Cui et al. 2020).

### 4.4 Limitations and Future Directions: Refining Predictive Models for Understory Ferns

This study comprehensively applied MaxEnt and GARP models to predict the potential distributions of five *Ophioglossum* species at the national scale in China for the first time. However, several limitations remain. For instance, the models did not fully exclude non‐growth areas such as water bodies and urban zones, which may lead to an overestimation of niche breadth. Additionally, some model parameters were predetermined, potentially affecting their expressive capacity (Sillero and Barbosa 2020). Furthermore, as understory herbs, the distribution of *Ophioglossum* species is influenced not only by abiotic factors like climate and soil but also by biotic interactions such as forest community structure and symbiotic relationships, which have not been integrated into the current models.

Future research could be improved in the following aspects: (1) optimizing model parameter settings and environmental layer processing to enhance the realism of predictions; (2) incorporating multiple GCMs to better capture the uncertainties in climate projections; (3) incorporating mechanistic processes such as population dynamics, dispersal limitations, and interspecific interactions to strengthen the ecological basis of the models; and (4) combining remote sensing and ground‐based monitoring data to achieve multi‐scale, dynamic distribution simulations and validation. These improvements will contribute to a more accurate assessment of how this rare medicinal plant group responds to global change and provide a more robust scientific basis for its conservation and sustainable use.

## 5 Conclusions

This study combined MaxEnt and GARP models to project and compare the potential distributions of five *Ophioglossum* species under current and future climates. Both models performed well (AUC > 0.84), but MaxEnt was chosen for future projections due to its greater computational efficiency and stability. Species distributions responded to temperature, precipitation, solar radiation, and soil conditions, with distinct environmental preferences reflecting niche specialization among these closely related species. Future changes in suitable habitat were generally limited, with no significant differences between the 2050s and 2070s, suggesting that climate change may not be the main driver of near‐term distribution shifts. Instead, habitat fragmentation, human disturbance, and species‐specific limits on reproduction and establishment are likely more critical to population survival. Conservation should thus prioritize current high‐suitability areas—such as Yunnan, Sichuan, Guizhou, and Hunan—by improving habitat connectivity and protecting key microhabitats to reduce human‐induced and natural threats. These results offer a scientific basis for conserving and managing *Ophioglossum* diversity. Future studies will refine model parameters and integrate population dynamics and dispersal constraints to enhance the ecological realism of predictions.

## Author Contributions

**Zhen‐Yan Pan:** formal analysis (equal), investigation (equal), writing – original draft (equal). **Jing‐Zhong Chen:** conceptualization (equal), supervision (equal), writing – original draft (equal), writing – review and editing (equal). **Fu‐Li Gong:** formal analysis (equal), investigation (equal). **Shun‐Chao Jia:** formal analysis (equal), investigation (equal). **Qing‐Wen Sun:** conceptualization (equal), funding acquisition (equal), supervision (equal), writing – review and editing (equal).

## Funding

Guizhou Provincial Basic Research Program (Natural Science), ZK [2024] ZD 075; Guizhou Provincial Foundation for Excellent Scholars Program, GCC [2023] 077; Guizhou Provincial Forestry Bureau 2026 Key Research Project: Research on in‐forest Cultivation Techniques for Traditional Chinese Medicinal Herbs, Qian‐Lin‐Ke‐He [2026] Key 006.

## Conflicts of Interest

The authors declare no conflicts of interest.

## Supporting information


**Figure S1:** Occurrence records for five *Ophioglossum* species before and after spatial filtering: (a) *O. petiolatum* , (b) *O. pedunculosum*, (c) *O. vulgatum* , (d) *O. reticulatum* , (e) *O. thermale* (Green circles = raw occurrence points; black circles = duplicate points removed during filtering; red triangles = final filtered occurrence points.)

**Figure S2:** Occurrence records for five *Ophioglossum* species after spatial filtering.

**Figure S3:** The AUC values of the MaxEnt model of the five closely related species of *Ophioglossum*: (a) *O. petiolatum* , (b) *O. pedunculosum*, (c) *O. vulgatum* , (d) *O. reticulatum* , and (e) *O. thermale*.

**Figure S4:** Importance ranking of environmental variables used for (a) *O. petiolatum* , (b) *O. pedunculosum*, (c) *O. vulgatum* , (d) *O. reticulatum* , and (e) *O. thermale* tested by the Jackknife‐cut method.

**Table S1:** Distribution points for the five *Ophioglossum* species before and after filtering.

**Table S2:** Environmental variables were considered for modeling the potentially suitable habitat of the five closely related species of *Ophioglossum* in China.

**Table S3:** Comparison of results between five geographically independent folds based on spatial blocking (blockCV) and the original random 75/25 split.

**Table S4:** Rule types selected via ENMeval‐based optimization for MaxEnt models of five closely related *Ophioglossum* species. (L: linear, Q: quadratic, H: hinge, P: product, T: threshold.)

**Table S5:** Selected rule types in GARP models for the five closely related *Ophioglossum* species.

**Table S6:** Complete list of environmental predictors used in the final models for five closely related species of the genus *Ophioglossum*.

**Table S7:** Ranges of key environmental variables across suitable habitats for the five *Ophioglossum* species.

## Data Availability

All occurrence data used in this study are publicly available from the Global Biodiversity Information Facility (GBIF, https://www.gbif.org/zh), Chinese Virtual Herbarium (CVH, https://www.cvh.ac.cn), and Plant Photo Bank of China (PPBC, http://ppbc.iplant.cn). The cleaned and spatially thinned occurrence datasets for the five *Ophioglossum* species, along with the final harmonized environmental layers (climate, soil, topography) used for modeling, all the required data are uploaded as Supporting Information.
