# Supplementary figures and images for: Environmental Adaptation Differences Are Key Factors Determining the Speciation and Future Adaptability of the Five Closely Related Species of the Genus Ophioglossum
Source: Ecol Evol. 2026 Mar 12;16(3):e73243. doi: 10.1002/ece3.73243 (PMC13093419; doi:10.1002/ece3.73243)

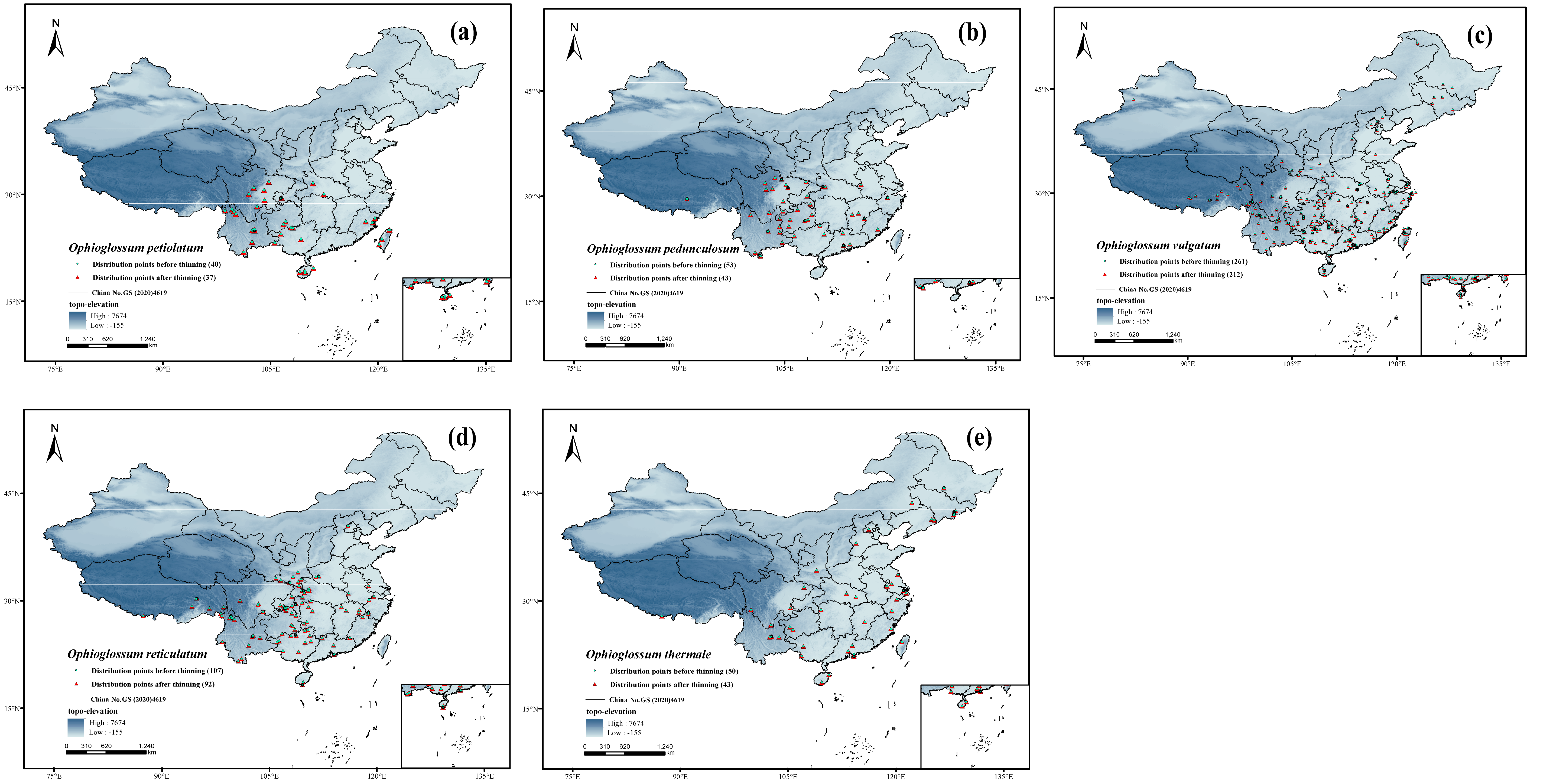

Supplement: Supplementary file 1 — Figure S1: Occurrence records for five Ophioglossum species before and after spatial filtering: (a) O. petiolatum , (b) O. pedunculosum, (c) O. vulgatum , (d) O. reticulatum , (e) O. thermale (Green circles = raw occurrence points; black circles = duplicate points removed during filtering; red triangles = final filtered occurrence points.) [file ECE3-16-e73243-s011.tif]

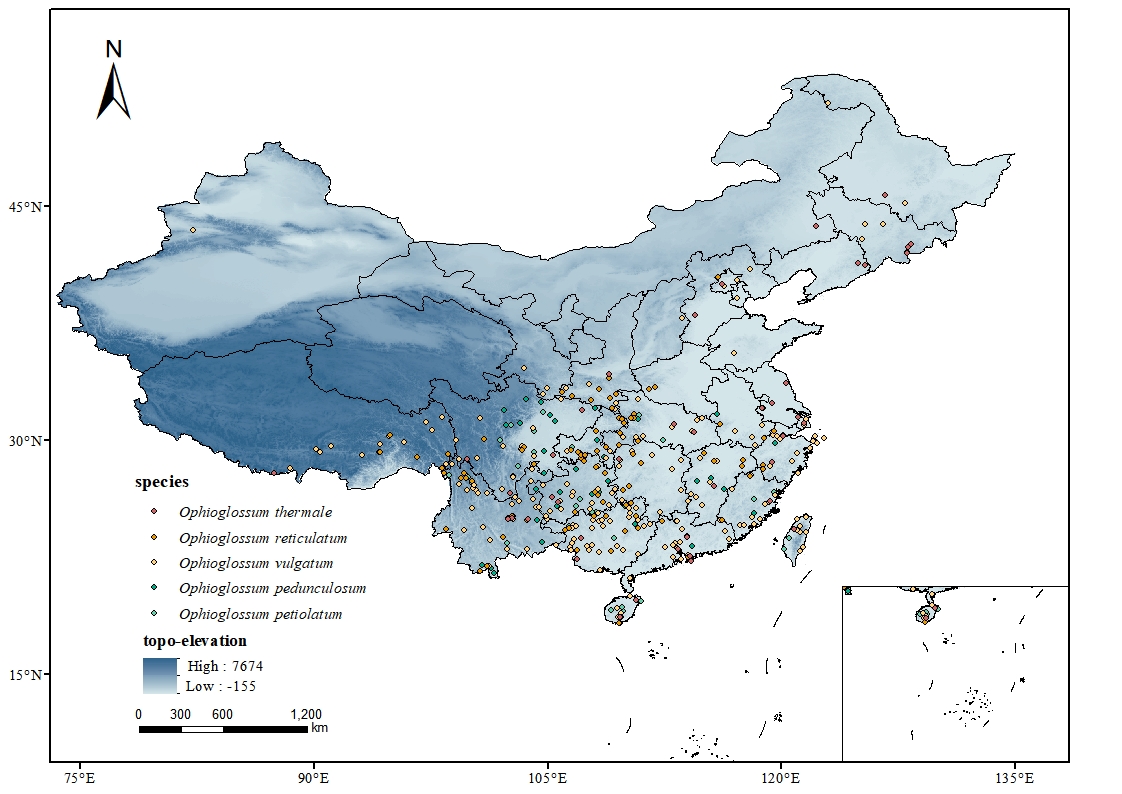

Supplement: Supplementary file 2 — Figure S2: Occurrence records for five Ophioglossum species after spatial filtering. [file ECE3-16-e73243-s002.jpg]

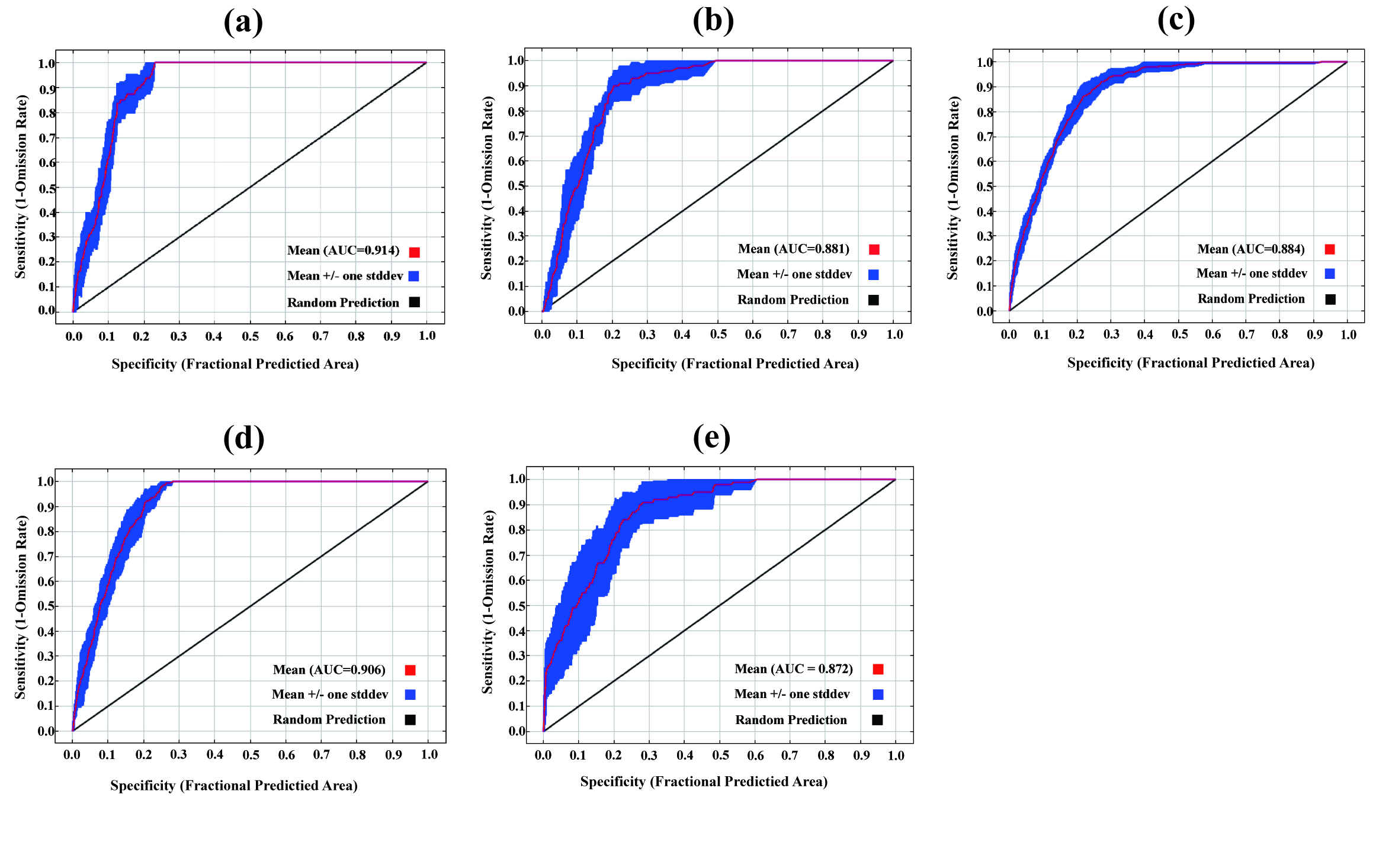

Supplement: Supplementary file 3 — Figure S3: The AUC values of the MaxEnt model of the five closely related species of Ophioglossum: (a) O. petiolatum , (b) O. pedunculosum, (c) O. vulgatum , (d) O. reticulatum , and (e) O. thermale. [file ECE3-16-e73243-s006.tif]

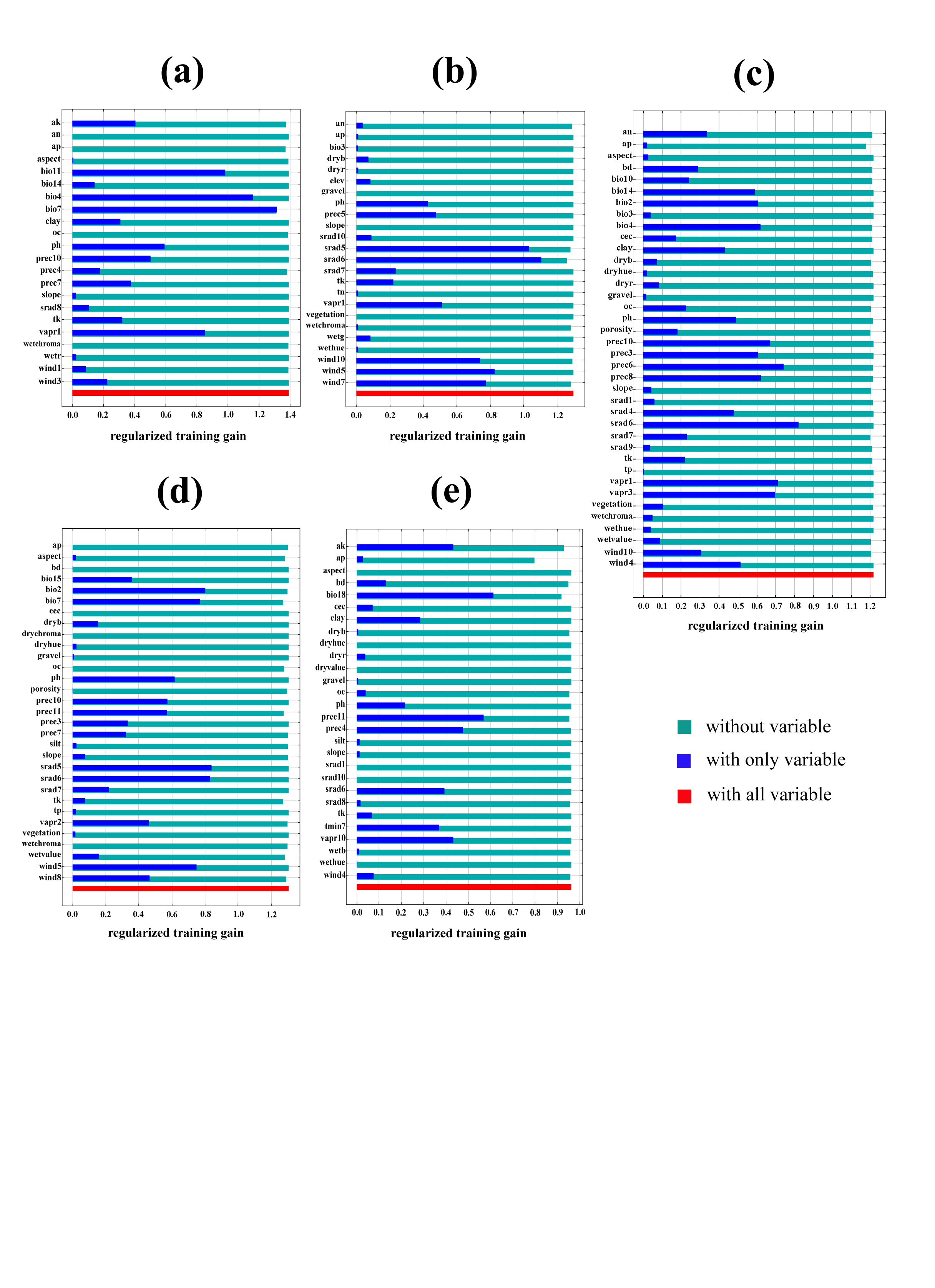

Supplement: Supplementary file 4 — Figure S4: Importance ranking of environmental variables used for (a) O. petiolatum , (b) O. pedunculosum, (c) O. vulgatum , (d) O. reticulatum , and (e) O. thermale tested by the Jackknife‐cut method. [file ECE3-16-e73243-s009.tif]
